# Repurposing FDA-approved sulphonamide carbonic anhydrase inhibitors for treatment of *Neisseria gonorrhoeae*

**DOI:** 10.1080/14756366.2021.1991336

**Published:** 2021-12-11

**Authors:** Nader S. Abutaleb, Ahmed E. M. Elhassanny, Alessio Nocentini, Chad S. Hewitt, Ahmed Elkashif, Bruce R. Cooper, Claudiu T. Supuran, Mohamed N. Seleem, Daniel P. Flaherty

**Affiliations:** a Department of Biomedical Sciences and Pathobiology, Virginia-Maryland College of Veterinary Medicine, Virginia Polytechnic Institute and State University, Blacksburg, VI, USA; b Department of NEUROFARBA, Section of Pharmaceutical and Nutraceutical Sciences, University of Florence, Polo Scientifico, Firenze, Italy; c Department of Medicinal Chemistry and Molecular Pharmacology, College of Pharmacy, Purdue University, West Lafayette, IN, USA; d Department of Comparative Pathobiology, College of Veterinary Medicine, Purdue University, West Lafayette, IN, USA; e Metabolite Profiling Facility, Bindley Bioscience Center, Purdue University, West Lafayette, Indiana, United States; f Center for Emerging, Zoonotic and Arthropod-borne Pathogens, Virginia Polytechnic Institute and State University, Blacksburg, VI, USA; g Purdue Institute for Drug Discovery, West Lafayette, IN, USA; h Purdue Institute of Inflammation, Immunology and Infectious Disease, West Lafayette, IN, USA

**Keywords:** Carbonic anhydrase inhibitors, *Neisseria gonorrhoeae*, antibiotics, drug repurposing

## Abstract

*Neisseria gonorrhoeae* is a high-priority pathogen of concern due to the growing prevalence of resistance development against approved antibiotics. Herein, we report the anti-gonococcal activity of ethoxzolamide, the FDA-approved human carbonic anhydrase inhibitor. Ethoxzolamide displayed an MIC_50,_ against a panel of *N. gonorrhoeae* isolates, of 0.125 µg/mL, 16-fold more potent than acetazolamide, although both molecules exhibited almost similar potency against the gonococcal carbonic anhydrase enzyme (NgCA) *in vitro*. Acetazolamide displayed an inhibition constant (*K*_i_) versus NgCA of 74 nM, while Ethoxzolamide^’^s *K*_i_ was estimated to 94 nM. Therefore, the increased anti-gonococcal potency of ethoxzolamide was attributed to its increased permeability in *N. gonorrhoeae* as compared to that of acetazolamide. Both drugs demonstrated bacteriostatic activity against *N. gonorrhoeae*, exhibited post-antibiotic effects up to 10 hours, and resistance was not observed against both. Taken together, these results indicate that acetazolamide and ethoxzolamide warrant further investigation for translation into effective anti-*N. gonorrhoeae* agents.

## 1. Introduction

*Neisseria gonorrhoeae*, a Gram-negative pathogen, and the causative agent of gonorrhoea, is an emerging super-pathogen that has seen a rapid rise in new cases worldwide over the past decade. The World Health Organisation (WHO) reported a surge of new cases of gonorrhoea from 78 million^1^ in 2012 to as many of 87 million new infections worldwide in 2016^2^. Moreover, the Centres for Disease Control and Prevention (CDC) reported a record of 583,000 new cases of drug-resistant gonorrhoea in the United States in 2018^3^.

The growing number of infections is only part of the problem. Equally concerning is the pathogen has demonstrated a keen ability to develop resistance to several classes of FDA-approved antibiotics. Global surveillance programs have identified alarming resistance rates to the available antibiotics such a β-lactams, tetracyclines, and quinolines^4^. As recently as 2017, 6–30% of *N. gonorrhoeae* isolates in the United States were resistant to ciprofloxacin and that number increased to 71–100% of isolates in other regions of the world^5^. In addition, resistance to the macrolide drug, azithromycin, has recently grown to greater than 33% in some regions of the world^5–7^. This emerging resistance was cited as one of the reasons the CDC removed azithromycin from the standard treatment guidelines for uncomplicated gonorrhoea, in 2020, leaving only ceftriaxone injection as the drug of choice^8^. However, although rare for the time being, isolated cases of treatment failure with ceftriaxone have been reported^9–11^. Due to the combination of increased rates of infection and prevalence of drug-resistant strains worldwide, both the WHO and CDC have classified drug-resistant *N. gonorrhoeae* within the highest threat level to public health^5^^,^^12^ and highlighted an urgent need to identify new molecular targets and chemical scaffolds to combat this pathogen. Otherwise, the world faces the real possibility of an untreatable gonococcal infection^13^.

One method that has the potential to "fast-track" discovery of new antibiotics is through a drug repurposing strategy^14^ because it accelerates the process of drug discovery and reduces the time to market^15^^,^^16^. To this end, there have been reports of FDA-approved carbonic anhydrase inhibitors (CAIs) with potency against several pathogens such as *Helicobacter pylori*^17–19^, *Neisseria* spp. ^20^^,^^21^, and *Mycobacterium tuberculosis*^22^. Other groups have reported the potential of human CAIs, or analogs thereof, against recombinant bacterial carbonic anhydrases^23–25^ including *Helicobacter pylori*^26^^,^^27^*, Vibrio cholerae*^28^*, Burkholderia spp*^29^^,^^30^, and *Streptococcus pneumoniae*^31^ to name a few. However, while many of these studies demonstrate activity against the bacterial carbonic anhydrases *in vitro*, they stop short of demonstrating antimicrobial efficacy against the pathogens. Also of note, while humans encode for only the α-carbonic anhydrase subfamily, bacteria have been found to encode for α-, β-, γ-, ι-subfamily carbonic anhydrases^29^^,^^32^^,^^33^. However, there is limited information about inhibition or ligand-bound structural data for inhibitors against non-α-class carbonic anhydrases.

**Figure 1.**
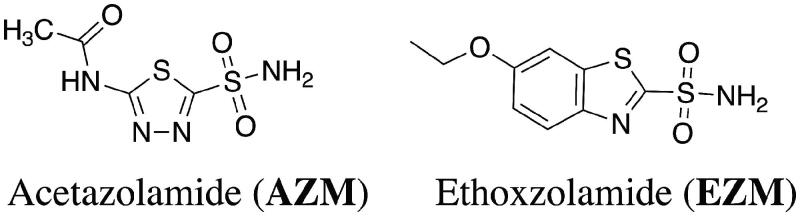
Structures of FDA-approved human carbonic anhydrase inhibitors acetazolamide and ethoxzolamide.

As part of a drug repurposing effort, our team has reported that the human CAI, acetazolamide (AZM), and the newly developed CAI scaffold displayed improved efficacy against vancomycin-resistant *Enterococcus* (VRE)^34–36^ and *N. gonorrhoeae*^37^ and are likely inhibiting essential bacterial α-carbonic anhydrases in each species. As an extension of that work, we report herein further studies comparing the human CAIs, acetazolamide (AZM) and ethoxzolamide (EZM) (Figure 1) for *in vitro* efficacy against *N. gonorrhoeae*.

Both AZM and EZM are approved for use as diuretics and used for treating glaucoma^38–41^. AZM is also prescribed as a chronic treatment for epilepsy^42^^,^^43^, symptoms associated with congestive heart failure^44^, and altitude sickness^45^^,^^46^. Additionally, AZM is investigated in various clinical trials (clinicaltrials.gov) that are active or in the recruitment phases, for the treatment of conditions such as ataxia (NCT04679389), sleep apnoea (NCT04726982), chronic obstructive pulmonary disorder (NCT04915365), and cancer (NCT03011671). Moreover, AZM is included in the WHO’s list of essential medicines due to its low cost, safe pharmacokinetic and toxicity profiles and overall efficacy^47^. Further, AZM is generally safe as a phase 1 clinical trial showed 89.5% of patients were able to tolerate >1 g/day dose for 6 months with 45% of patients tolerating up to 4 g/day over the same time period^48^^,^^49^. The drug is often dosed chronically to patients at >1 g/day^49–51^, is orally bioavailable with a plasma elimination half-life of 4–6 h in adults after 250 mg oral dose^45^, and 100% of the drug is excreted in the urine with no metabolites^52–54^. It has also been shown that due to the expression of human carbonic anhydrase isoforms I and II in erythrocytes, AZM partitions readily into the red blood cells portion of blood^55^^,^^56^. This effectively forms a sink that sequesters the drug and doubles the elimination half-life (∼12 h) when the whole blood concentration is quantified^45^.

Concerning EZM, there is considerably less publicly available information regarding safety, tolerability, and pharmacokinetics. If dosed orally, it is commonly prescribed at 125 mg every 6 h and has a plasma half-life of 6 h in humans^57^. Unlike AZM, only 40% of EZM appears unchanged in the urine with the rest of the molecule being *O*-dealkylated followed by subsequent glucuronidation at the free phenol^39^.

While previous literature has demonstrated that AZM and EZM have activity against *N. gonorrhoeae*^20^^,^^21^, it has stopped short of further elucidating the antimicrobial properties for these molecules against the pathogen. In the case of EZM, while Sanders and Maren reported anti-*Neisseria* activity^20^, they only provided ranges of MICs and not a full study on the drug's antimicrobial properties. Our recent work further investigated AZM alongside new CAI analogs^37^, but there is still more to be learned from how AZM and EZM affect *N. gonorrhoeae* beyond standard MIC measurements. To this end, we present additional data for the antimicrobial properties of AZM and EZM including *in vitro* activity versus the proposed intracellular target, MICs versus a panel of clinical *N. gonorrhoeae* strains, antibiotic efficacy with regards to killing kinetics, post-antibiotic effects, the frequency of resistance development and bacterial accumulation in *N. gonorrhoeae* are all described.

## 2. Materials and methods

### 2.1. Bacterial strains, media and chemicals

*N. gonorrhoeae* strains (Table S1) used in the study were clinical isolates obtained from the CDC and the American Type Culture Collection (ATCC) (Manassas, VA, USA). Drugs used in this study were purchased from chemical vendors: AZM (Alfa Aesar, Tewksbury, MA, USA), EZM and tetracycline (Sigma-Aldrich, Saint Louis, MO, USA), azithromycin and ceftriaxone (TCI America, Portland, OR, USA). Media and reagents were purchased commercially: brucella broth, GC medium base, IsoVitaleX, bovine haemoglobin, and chocolate II agar plates (Becton, Dickinson and Company, Cockeysville, MD, USA), yeast extract and dextrose (Fisher Bioreagents, Fairlawn, NJ, USA), protease peptone (Oxoid, Lenexa, KS, USA), haematin, pyridoxal, and nicotinamide adenine dinucleotide (NAD) (Chem-Impex International, Wood Dale, IL, USA), corn starch (Spectrum Chemical MFG, Gardena, CA, USA), monobasic potassium phosphate and dibasic potassium phosphate (Macron chemicals, Centre Valley, PA, USA), and sodium chloride (Fisher Scientific, Fail Lawn, NJ, USA), and phosphate-buffered saline (PBS) (Corning, Manassas, VA, USA).

**Table 1.** MICs for AZM and **EZM** against a panel of *N. gonorrhoeae* clinical isolates.

	MIC (µg/mL)			
*N. gonorrhoeae* strains	AZM^a^	EZM	AZI	CEF
CDC 165	4	0.25	1	0.06
CDC 166	4	0.125	1	0.125
CDC 167	2	0.25	4	0.015
CDC 168	4	0.125	1	0.125
CDC 169	4	0.125	1	0.125
CDC 173	1	0.06	1	0.125
CDC 178	2	0.125	1	0.03
CDC 179	4	0.125	4	0.008
CDC 181	2	0.25	>64	0.015
CDC 182	4	0.125	1	0.03
CDC 183	2	0.06	1	0.03
CDC 184	2	0.06	0.5	0.06
CDC 186	2	0.125	0.5	0.03
CDC 187	2	0.125	2	0.015
CDC 197	4	0.25	2	0.015
CDC 202	4	0.25	16	0.008
CDC 211	1	0.25	2	0.03
ATCC700825	0.5	0.125	0.5	0.008
WHO-V	0.5	0.015	16	0.008
WHO-W	2	0.25	0.125	0.06
WHO-X	1	0.25	0.25	0.5
WHO-Z	4	0.5	2	0.5
**^b^MIC_50_**	2	0.125	1	0.03
**^c^MIC_90_**	4	0.25	16	0.125

AZM: acetazolamide; EZM: ethoxzolamide; AZI: azithromycin; CEF: ceftriaxone. ^a^Previously reported by Hewitt et al^37^. ^b^MIC_50_: minimum inhibitory concentration at which the drug inhibited 50% of the tested strains. ^c^MIC_90_: minimum inhibitory concentration at which the drug inhibited 90% of the tested strains.

### 2.2. Antibacterial activity of AZM and EZM against N. gonorrhoeae strains

The minimum inhibitory concentrations (MICs) of compounds were carried out as described previousy^37^^,^^58–60^. Briefly, *N. gonorrhoeae* strains were grown overnight on chocolate II agar plates. Bacterial cells were then suspended in PBS to achieve turbidity equivalent to a 1.0 McFarland standard which was diluted in Brucella broth supplemented with yeast extract, dextrose, protease-peptone, NAD, pyridoxal, haematin and IsoVitaleX to reach a bacterial count of about 1 × 10^6^ CFU/mL. Drugs were added and serially diluted along with the plates. Media containing bacteria (without test agents) were included in the assays as a control. Plates were then incubated in the ambient air, and in presence of 5% CO_2_ for 24 h at 37 °C before recording the MICs as observed visually. MICs reported are the minimum concentrations of drugs that completely inhibited the visual growth of bacteria.

### 2.3. Carbonic anhydrase CO_2_ hydration catalytic assay and K_i_ determination

The assay was performed according to previously published protocols^37^^,^^61–65^. Recombinant *N. gonorrhoeae* carbonic anhydrase (NgCA) was produced as previously described^37^. Recombinant human carbonic anhydrase (hCA) I and hCA II were purchased from Millipore Sigma (hCA I Catalog# C4396-5MG; hCA II Catalog # C6624-500UG). *K*_i_ values were determined from inputting the IC_50_ values into the Cheng-Prusoff equation^66^ for *K*_i_ from catalytic inhibition constants.

### 2.4. Killing kinetics assay

To determine the mode of the killing of AZM and EZM, a standard time-kill kinetics assay was performed against *N. gonorrhoeae* ATCC 700825 as described previously^67–69^. *N. gonorrhoeae* was grown in GC broth supplemented with IsoVitaleX to logarithmic phase and further diluted to reach an initial inoculum of ∼10^6^ CFU/mL. AZM, EZM, azithromycin, and ceftriaxone were then added (at 10× MIC in triplicates), and further incubated in the ambient air at 37 °C for 24 h. Bacteria exposed to DMSO (solvent of drugs) served as a negative control. An aliquot from each treatment was collected after the corresponding times of incubation and subsequently serially diluted and plated onto chocolate II agar plates. Plates were incubated for 24 h at 37 °C before viable CFU/mL was determined.

### 2.5. Accumulation of AZM and EZM inside N. gonorrhoeae cells

#### 2.5.1. Sample preparation

The accumulation assay was performed as described previously^70^^,^^71^. An overnight culture of *N. gonorrhoeae* ATCC 700825 was diluted 1:100 in GC broth supplemented with IsoVitaleX, and grown at 37 °C with shaking for 12 h. The bacteria were pelleted, washed once in PBS and then resuspended in fresh PBS, aliquoted into sixteen 1.7 ml Eppendorf tubes (4 tubes for each test agent), diluted, and counted. AZM, **EZM,** and tetracycline (positive control) were added at a concentration of 50 μM. DMSO was included as a negative control. Samples were then incubated at 37 °C with shaking for either 10 min., 30 min., 1 h, or 2 h. After incubation, 800 μL of the cultures were carefully layered on 700 μL of silicone oil mixture (AR20/High Temperature (9:1)). Bacteria were pelleted through the oil by centrifuging at 13,000 r.c.f. for 2 min. at room temperature. The supernatant and oil were then removed by pipetting. Afterwards, samples were lysed by resuspending the pellet in 200 μL of sterile distilled water, and then they were subjected to four freeze-thaw cycles (three minutes in liquid nitrogen followed by three minutes in a water bath at 65 °C). The lysates were pelleted and the supernatant was collected. The debris was re-suspended in 100 μL of methanol and pelleted as before. The supernatants were removed and combined with the previous supernatants collected. The remaining debris was removed by centrifuging. Samples (50 μL) from each replicate were diluted 10-fold in a solution of 25% water and 75% methanol. Sample solutions were sonicated for 5 min, centrifuged at 16,000 g for 8 min, then the supernatants were transferred to HPLC vials.

#### 2.5.2. HPLC/MS-MS analysis

Samples were quantitated for AZM, EZM, and tetracycline by HPLC/MS-MS. Separations were performed on an Agilent Rapid Res 1200 HPLC system using an Agilent Zorbax XDB-C18 (2.1 × 50 mm, 3.5 μm) column. Mobile phase A was water with 0.1% formic acid and mobile phase B was acetonitrile with 0.1% formic acid. Initial conditions were 90:10 A:B, followed by a linear gradient to 10:95 at 10 min. Column re-equilibration was performed by returning to 90:10 A:B at 11 min and held until 16 min. The column flow rate was 0.3 ml/min. Retention times for AZM, EZM, and tetracycline were 1.5, 7.0, and 4.0 min, respectively.

Analytes were quantified by MS/MS utilising an Agilent 6460 triple quadrupole mass spectrometer with positive electrospray ionisation (ESI). Quantitation was based on Multiple Reaction Monitoring (MRM). AZM was detected with a transition of 223.0–181.0 (quantifier) and 163.9 (qualifier), with collision energies (CE) of 10 V and 20 V, respectively. EZM was detected with a transition of 258.9–178.0 (quantifier) and 150.0 (qualifier), with CE of 15 V and 20 V, respectively. Tetracycline was detected with a transition of 445.1–410.1 (quantifier) and 154.0 (qualifier), with CE of 20 V and 25 V, respectively. Fragment energy of 145 V and a dwell time of 200 ms were used. All data were collected and analysed with Agilent MassHunter B0.03 software. Quantitation was based on 5-point standard curves, with concentration ranges from 3 to 1,700 ng/mL. Correlation coefficients of >0.9999 were obtained.

### 2.6. Post-antibiotic effect of AZM and EZM

The post-antibiotic effect (PAE) of AZM and EZM was tested against two *N. gonorrhoeae* strains (CDC 181, and CDC 186) following the procedure previously described^72–74^. Briefly, *N. gonorrhoeae* strains were grown in brucella supplemented broth to logarithmic phase and further diluted to reach an initial inoculum of about 1 × 10^6^ CFU/mL. AZM, **EZM,** or azithromycin (10 × MIC, in triplicates) were added to bacteria and incubated at 37 °C with agitation under ambient air conditions for one hour. DMSO was included as growth control. Thereafter, drugs were removed by diluting each tube 1:1000 in fresh brucella supplemented broth, and tubes were incubated as previously described for 12 h. Samples were collected from each tube every two hours, serially diluted, and plated on GC II agar plates. The PAE was calculated using this equation: PAE = T − C, where T is the time taken by the bacterial culture treated with the drug to increase by one log_10_, while C is the time required for the negative control (DMSO) to increase by one log_10_.

### 2.7. Frequency of spontaneous mutation

*N. gonorrhoeae* CDC 202 was tested against AZM, **EZM,** or rifampin for a single-step mutation assay as previously described^75–77^. Briefly, drugs were mixed with GC agar supplemented with IsoVitaleX at the concentrations of 5× and 10× MIC. Plates were then prepared and allowed to dry at room temperature. A bacterial suspension (∼10^9^ CFU/mL) was prepared and spread over the plates containing the drugs tested. Plates were incubated at 37 °C under ambient air conditions for 48 h before determining the bacterial CFU.

## 3. Results

### 3.1. Anti-gonococcal activity of AZM and EZM

As depicted in Table 1, both AZM and EZM were tested for antimicrobial activity against a panel of 22 *N. gonorrhoeae* clinical isolates including WHO reference isolates. In general, EZM outperformed AZM across the panel tested with MIC values ranging from 0.06 to 0.5 µg/mL. EZM inhibited 50% of the isolates tested (MIC_50_) at the concentration of 0.125 µg/mL and 90% of the tested isolates (MIC_90_) at 0.25 µg/mL. This equated to 16-fold better potency as compared to AZM, which exhibited MIC_50_ and MIC_90_ values of 2 µg/mL and 4 µg/mL, respectively. Azithromycin (AZI) inhibited strains tested at concentrations ranging from 0.125 µg/mL to 4 µg/mL (with exception of two strains that exhibited high-level resistance to AZI with MIC values of >64 µg/mL), with MIC_50_ and MIC_90_ values of 1 µg/mL and 16 µg/mL, respectively. Interestingly, EZM was superior to AZI against the majority of the tested strains at approximately 4- to 64-fold greater potency in MIC_50_ over AZI. Additionally, AZM and EZM maintained their activity against azithromycin-resistant *N. gonorrhoeae* isolates (CDC 181, CDC 202, and WHO-V), indicating no cross-resistance between the CAIs AZM and EZM, and AZI.

However, EZM was not as potent against the panel tested as compared to the current standard drug of choice for gonorrhoea, ceftriaxone (CEF), which displayed an MIC_50_ value of 0.03 µg/mL and MIC_90_ value of 0.125 µg/mL, equating to 4- and 2-fold less potency for the two metrics. Nonetheless, these results indicate that EZM maintains sub-1 µg/mL antimicrobial activity, outperforms AZI, and is comparable to CEF although slightly less potent.

### 3.2. Intracellular target identification

Our group and others have reported previously on CAIs potential in targeting *N. gonorrhoeae* and suggest that the molecules could be targeting the intracellular *N. gonorrhoeae* α-carbonic anhydrase (NgCA)^20^^,^^37^. EZM is also a potent CAI of human carbonic anhydrases and likely maintains similar activity against NgCA. Thus, we performed an assay to test for *N. gonorrhoeae* susceptibility in CO_2_ saturating conditions. Since CO_2_ is the natural substrate of carbonic anhydrases, at higher levels in the bacterial culture, it will outcompete with the inhibitor for the substrate-binding site^20^^,^^78^. Consequently, in these conditions, if the inhibitor is targeting the NgCA intracellularly, the bacteria should display reduced susceptibility. Similar to AZM, it was observed that *N. gonorrhoeae* strains displayed reduced susceptibility to EZM in presence of 5% CO_2_ (representative strains shown in Table 2, and data for all strains tested shown in Table S2). As a control the same assay was performed with azithromycin and ceftriaxone, two molecules with mechanisms of action not linked to CO_2_ metabolism, to ensure no unintended resistance was observed that may confound interpretation of the results observed for the CAIs. For both molecules, there was no observed change in susceptibility to *N. gonorrhoeae* between the normal and CO_2_ conditions providing confidence that the reduced susceptibility in the case of the CAIs could indeed be due to NgCA CO_2_ saturation. While these results suggest a high probability that NgCA is the intracellular target for CAIs further work is being performed to definitively link CAI engagement with NgCA.

**Table 2.** MICs of molecules under normal and CO_2_ conditions.

Test agent	*N. gonorrhoeae* CDC 181^a^		*N. gonorrhoeae* CDC 178^a^		*N. gonorrhoeae* WHO-X^a^	
	Normal^b^	CO_2_^c^	Normal^b^	CO_2_^c^	Normal^b^	CO_2_^c^
AZM	4^d^	>64^d^	2^d^	>64^d^	1	>64
EZM	0.25	64	0.125	>64	0.25	>64
AZI	>64^d^	>64^d^	2^d^	2^d^	0.25	0.25
CEF	0.015	0.015	0.03	0.03	0.5	0.5

AZM: acetazolamide; EZM: ethoxzolamide; AZI: azithromycin; CEF: ceftriaxone. ^a^MIC values in µg/mL. ^b^indicates standard conditions in ambient air. ^c^indicates incubation in presence of 5% CO_2_. ^d^data reported by Hewitt et al.^37^.

### 3.3. In vitro inhibition of carbonic anhydrases

EZM exhibited 16-fold improved MIC_50_ and MIC_90_ than AZM towards *N. gonorrhoeae*. Thus, we sought to assess whether the increased potency for EZM was a function of differing *in vitro* potencies against NgCA. Therefore, we collected inhibition constant (*K*_i_) data in a CO_2_ hydration assay as previously described^37^. Interestingly, AZM displayed greater potency in terms of *K*_i_ versus NgCA (*K*_i_ = 74 nM) compared to EZM (*K*_i_ = 94 nM) by approximately 1.2-fold (Table 3). Consequently, the large difference in anti-gonococcal potency between EZM and AZM cannot be attributed to the *in vitro* inhibition of NgCA for AZM and EZM. For comparison, the inhibitory constants are also provided for both molecules against two highly expressed human carbonic anhydrase isoforms, hCA I and hCA II. These isoforms, like NgCA, are also α-carbonic anhydrase family enzymes and are relevant for the distribution and pharmacokinetics of carbonic anhydrase inhibitors as they are prevalent in the red blood cells of humans^55^^,^^56^. EZM potency favoured the human isoforms over NgCA, by more than 10-fold for hCA II and about 4-fold for hCA I, while AZM was more potent towards NgCA over hCA I but not hCA II.

**Table 3.** Inhibitory constants for AZM and **EZM** against NgCA and hCAs

	CO_2_ Hydration *K*_i_ (nM)		
	NgCA	hCA I	hCA II
AZM^a^	74 ± 3^b^	250 ± 11^b^	13.0 ± 0.8^b^
EZM	94 ± 7	25 ± 4	8.0 ± 1.2

AZM: acetazolamide; EZM: ethoxzolamide.

^a^Catalytic CO_2_ hydration assay *K*_i_ determined from the mean of one experiment performed in triplicate and IC_50_ values entered into Cheng-Prusoff equation. Values reported are ± standard error of the mean.

^b^Previously reported by Hewitt et al.^37^.

### 3.4. Molecule accumulation in N. gonorrhoeae

Since NgCA inhibition did not provide further details about superiority in the anti-gonococcal activity for EZM over AZM, we sought to investigate whether the molecules exhibited differences in accumulation within this Gram-negative pathogen and if this factor could account for EZM’s superior anti-microbial activity. Therefore, we set out to quantify the accumulation levels of both AZM and EZM as well as tetracycline (positive control) using the procedure described by Richter et al.^70^.

**Figure 2.**
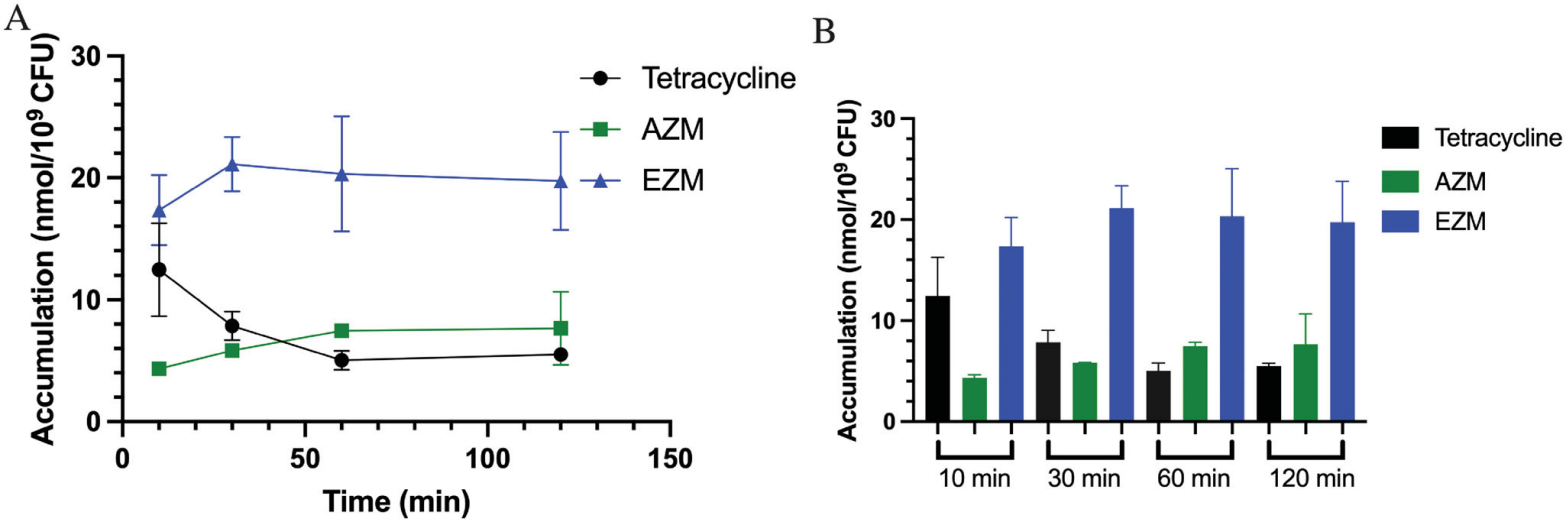
Drug accumulation in *N. gonorrhoeae* ATCC700825 over 120 min at pH 7.4. (A) Plot of accumulation in nmol/10^9^ CFU over 120 min after treatment with AZM, EZM, and tetracycline. (B) Accumulation of each drug at each time point in ng/10^9^ CFUs. Each drug was dosed at a concentration of 50 µM into 1 ml tubes containing 10^9^ CFU of *N. gonorrhoeae*.

Quantitation of the molecules indicated that EZM rapidly accumulated and peaked at 21.1 ± 2.2 nmol/10^9^ CFU at the 30-min time-point then maintained a concentration around 20 nmol/10^9^ CFU for the remainder of the 120-min duration. This accumulation equated to 3.6-fold greater accumulation compared to AZM (5.8 ± 0.1 nmol/10^9^ CFU) at 30 min, while the concentration for AZM steadily increased and peaked at 7.7 nmol/10^9^ CFU at the 120-min time point (Figure 2 and tabular data in Table S3). Given that the total dose of molecule equated to 50 nmol/10^9^ CFUs in each sample the amount of EZM detected at the 30-min time point indicated approximately 40% of the total EZM in solution accumulated within the bacterial cell compared to approximately 11% of the total AZM in solution at 30 min. Tetracycline was used as a positive control and exhibited rapid accumulation followed by a steady decline in molecule retention over the course of 120 min. Given that EZM ranged from approximately 2.5 to 3.5-fold greater accumulation within *N. gonorrhoeae* ATCC700825, it could be reasonably deduced that the improved accumulation of EZM is a primary factor for its increased potency over AZM. Future studies will evaluate the difference in accumulation in strains where the MIC values exhibited a wider gap in potency between the two compounds to assess if there are greater differences in permeability, and retention, among these strains. Nonetheless, there appear to be physicochemical properties intrinsic to EZM that provide this molecule with favourable accumulation over AZM.

### 3.5. Time-kill and post-antibiotic effects for carbonic anhydrase inhibitors

**Figure 3.**
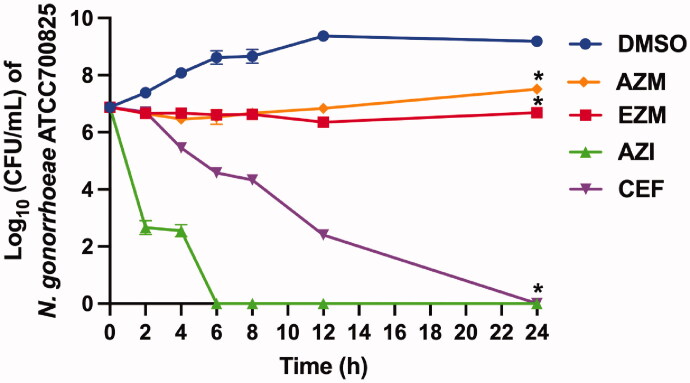
Time-kill assay of CAIs, azithromycin (AZI), and ceftriaxone (CEF) (*n* = 4, at 10 × MIC) against *N. gonorrhoeae* ATCC 700825. DMSO (vehicle) served as a negative control. The error bars represent standard deviation values for each test agent studied. The data were analysed via a two-way ANOVA with post-hoc Dunnett's test for multiple comparisons. An asterisk (*) indicates a statistically significant difference (*P* < 0.05) between treatment with drugs compared to DMSO treatment (negative control).

The CAIs were assessed in killing kinetics assay and evaluated for post-antibiotic effects to further understand their antimicrobial properties. Recently we showed that AZM and analogs exhibit bacteriostatic effects against *N. gonorrhoeae*^37^. Unsurprisingly, the same bacteriostatic effect was observed for EZM (Figure 3). Yet, both AZM and EZM were found to significantly reduce *N. gonorrhoeae* burden as compared to DMSO (negative control). After 24 h, EZM reduced *N. gonorrhoeae* burden by 0.4-log_10_ units. In addition, where at the 24-h time point the molecule provided approximately 2.5-log_10_-reduction in *N. gonorrhoeae* load compared to the DMSO-treated control. Azithromycin and ceftriaxone each exhibited bactericidal activity consistent with previous reports^58^^,^^79^.

Next, the post-antibiotic effect (PAE), the period after complete removal of the drug in which an antibiotic effect is still observed, was determined for the CAIs and azithromycin. The PAE is important data that, in combination with *in vivo* pharmacokinetics, can inform the proper dosing regimen^80^^,^^81^. PAE was determined as previously described^72^^,^^73^^,^^82^. AZM displayed an observed PAE ranging from 2 to 4 h against *N. gonorrhoeae* strains tested while EZM exhibited a long PAE of at least 10 h against all the strains tested (Table 4). EZM also slightly outperformed azithromycin (PAE = 8 h) in its PAE. These results are consistent with the permeability data that suggests EZM maintains a high intracellular concentration for at least 2 h with little decline over that period. Although the accumulation assay currently has not been extended out to the 10-h timepoint tested in the PAE assay, the PAE data suggests EZM maintains appreciable levels of molecule inside the cell to maintain the antimicrobial effect for this period.

**Table 4.** Post-antibiotic effect of molecules against *N. gonorrhoeae*.

	Post-antibiotic effect (hours)		
Strain	AZM	EZM	AZI
CDC 181	2	10	8
CDC 186	4	10	8

AZM: acetazolamide; EZM: ethoxzolamide; AZI: azithromycin. Drugs tested at 10 × MIC.

### 3.6. Frequency of spontaneous mutation

Given the promising results of CAIs, AZM and EZM, we sought to investigate the likelihood that *N. gonorrhoeae* will develop resistance to CAIs using the single-step spontaneous mutation assay and the results are presented in Table 5. No resistant mutants were isolated at concentrations of 5× and 10 × MIC in the presence of a high inoculum (5.42 × 10^9^ CFU/mL) of *N. gonorrhoeae* CDC 202, indicating a frequency of mutation of < 5.42 × 1 0 ^−9^. This result is comparable to that of azithromycin that exhibits low mutation frequency, as reported earlier^83^. The frequency of mutation of rifampin (positive control) was higher (1.1–1.5 × 1.0^−7^), as previously reported^58–60^.

**Table 5.** Spontaneous mutation frequencies of AZM, **EZM,** and rifampicin against *N. gonorrhoeae 202.*

Drugs	5 × MIC	10 × MIC
AZM	<5.42 × 10^−9^	<5.42 × 10^−9^
EZM	<5.42 × 10^−9^	<5.42 × 10^−9^
Rifampicin	1.49 × 10^−7^	1.14 × 10^−7^

## 4. Discussion

In previous work, our group has reported the efficacy of AZM and AZM-based analogs against *N. gonorrhoeae*. In the present work, we have extended the effort to investigate another FDA-approved CAI, EZM, that displays antimicrobial activity against *N. gonorrhoeae.* While both AZM and EZM have been reported previously to possess some level of anti-gonococcal activity^20^, the exact MIC values had not been reported. Thus, we set out to characterise the EZM’s antimicrobial activity against *N. gonorrhoeae* clinical isolates and compare it to AZM. The results presented herein show that EZM possessed superior antimicrobial potency towards *N. gonorrhoeae* compared to both AZM and azithromycin. For example, across a panel of 22 *N. gonorrhoeae* isolates, EZM’s MIC_50_ and MIC_90_ were 16-fold more potent than AZM and 8–64-fold more potent than AZI. The MIC_50_ for EZM was roughly 4-fold weaker than the current therapeutic option for gonorrhoea, ceftriaxone. Accordingly, this data suggests that EZM is comparable or better in terms of antimicrobial activity compared to the current, or former, treatments for the pathogen.

Since EZM was significantly more potent than AZM, even though they are hypothesised to be inhibiting at the same intracellular target, we sought to investigate the reason for this divergence in antimicrobial potency. We first evaluated both CAIs *in vitro* against NgCA to determine if a difference in biochemical inhibition may provide clues to EZM’s superior antimicrobial activity. Both CAIs are generally regarded to be essentially equipotent against human α-CA isoforms, with *K*_i_ values ranging from 0.8 to 250 nM across a panel of 12 human isoforms^84^. We found that AZM is slightly more potent against NgCA compared to EZM (74 and 94 nM, respectively). Therefore, we conclude the increase in antimicrobial activity against *N. gonorrhoeae* strains for EZM could not be driven by binding to the proposed target, NgCA. We are continuing to pursue target engagement studies in the bacterium, beyond the culture in CO_2_ conditions reported here, to definitively identify NgCA as the intracellular target. However, at this point, we cannot rule out the possibility of a second intracellular target that may be more susceptible to EZM inhibition than AZM, which in turn, may increase the susceptibility of the pathogen to the drug. Additionally, it should be noted that the CAI sulphonamides (AZM and EZM) are structurally related to sulfa-drugs that inhibit dihydropteroate synthase (DHPS), which previously were effective anti-gonorrheal agents^85^. However, as noted previously by our group, these sulfa-drugs do not have activity against the *N. gonorrhoeae* strains tested^37^. Therefore, neither AZM nor EZM are likely inhibiting DHPS as part of their antimicrobial mechanism of action. Nevertheless, further work is scheduled to further elucidate the mechanism of action of CAIs against *N. gonorrhoeae*.

**Figure 4.**
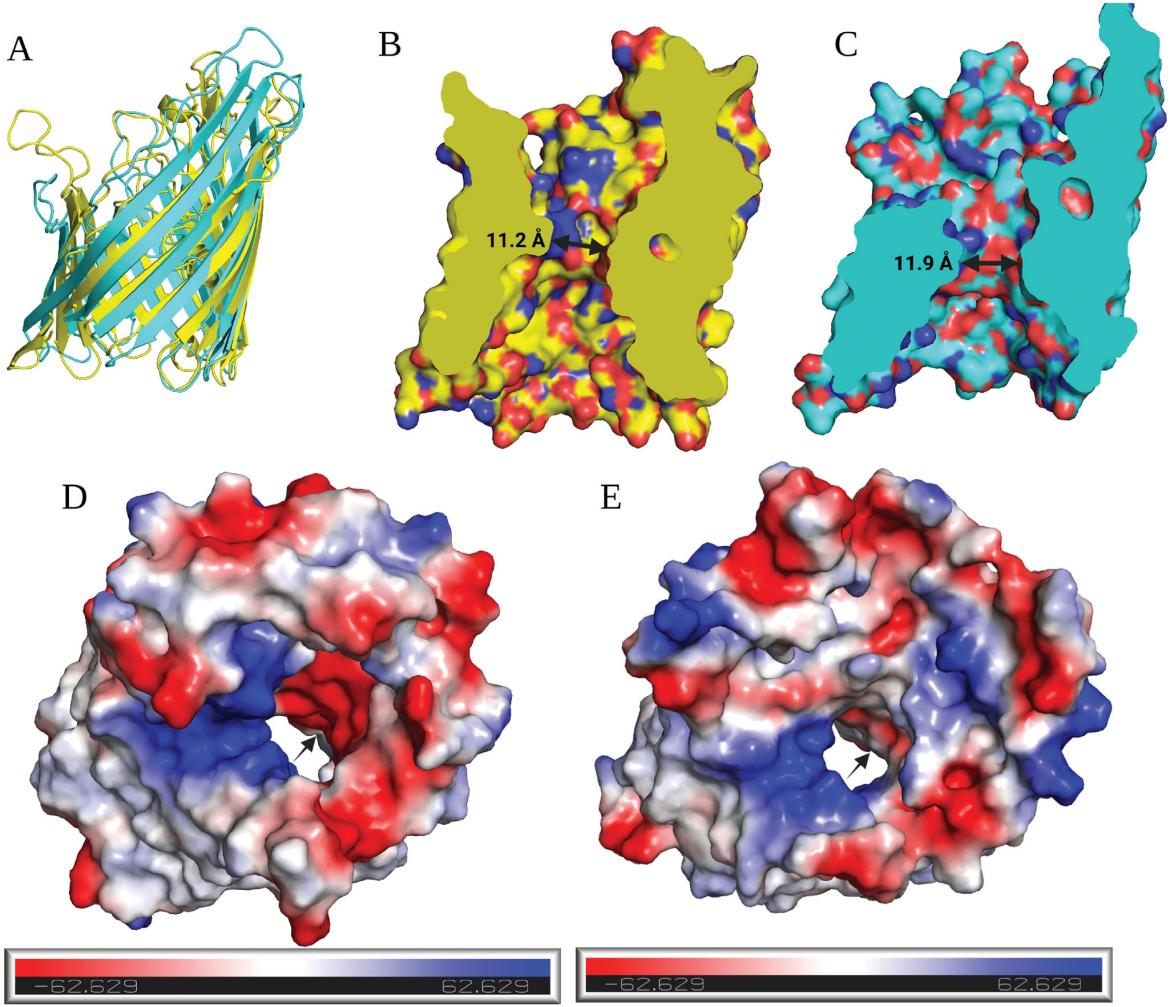
Comparison of *E. coli* porin OmpF and *N. gonorrhoeae* porin PorB. (A) Overlay of *E. coli* porin OmpF (yellow, PDB: 1OPF) and *N. gonorrhoeae* (cyan, PDB: 4AUI). (B) Side view cross-section of OmpF, porin constriction point depicted by a double-headed arrow with diameter of 11.2 Å. (C) Side view cross-section of PorB, porin constriction point depicted by the double-headed arrow with a diameter of 11.9 Å. (D) Surface electrostatic potential of OmpF, blue = positive charge and red = negative charge, arrow points to anionic surface patch. (E) Surface electrostatic potential of PorB, blue = positive charge and red = negative charge, arrow points to anionic surface patch. Overlays, surface representations, and electrostatic potentials are calculated in PyMol version 2.3.3 (Schrödinger, LLC). This figure was created on BioRender.com.

Gram-negative pathogens are notoriously difficult to treat due to the presence of the outer membrane that provides a barrier to small molecules, and the presence of efflux pumps that expel molecules from the cell. Thus, much effort has been applied recently to elucidate the physicochemical properties of molecules that influence permeability, and retention, within Gram-negative bacteria^70^^,^^86–88^. Richter et al. have established eNTRy rules for accumulation within *Escherichia coli* that suggest molecules with globularity < 0.25 (generally flat in nature), low flexibility (reduced number of rotatable bonds; < 5), and presence of a primary amine (positive charge in nature) all can increase accumulation within *E. coli*^70^. However, these rules were defined using *E. coli* as the model system and may not be fully applicable to *N. gonorrhoeae* accumulation as we described in our prior publication^37^. Nonetheless, AZM and EZM do satisfy two of the criteria suggested by the eNTRy rules, those being of low globularity and low flexibility. Conversely, the CAIs do not possess a primary amine, or any positively charged group, a structural component that had a significant positive influence on accumulation within *E. coli*. Yet, they still exhibit antimicrobial potency against the bacterium suggesting intracellular accumulation is achieved. Therefore, we turned to investigate the role bacterial accumulation may have on both AZM and EZM concerning the anti-gonococcal activity. Previous analysis by O’Shea and Moser on the physicochemical properties of antimicrobial compounds available on the market suggests that Gram-negative active molecules generally possess lower polar surface area and fewer hydrogen bond donors and acceptors^89^. They also suggest that overall charge, or lack thereof, maybe a key element that influences their anti-Gram-negative activity. This was taken a step further by Richter et al. when they proposed the aforementioned eNTRy rules for Gram-negative accumulation and note the importance of a primary amine in the driving accumulation within the *E. coli* cell^70^. These rules were developed and rationalised in *E. coli* that expresses the non-specific porin OmpF. *N. gonorrhoeae* contain two porins, PorA and PorB, that are structurally similar to OmpF (Figure 4A, overlay of OmpF; PDB: 1OPF^90^ and PorB; PDB: 4AUI^91^) yet possess some key differences. For example, the general pore size for PorB compared to OmpF is relatively similar (Figure 4B,C)^91^, with a measured distance at the constriction point for OmpF of 11.2 Å and 11.9 Å for PorB. Thus, we expect the globularity and rigidity metrics from the eNTRy rule to still apply to *N. gonorrhoeae*.

However, the charge distributions at the constriction points are quite different. As shown for OmpF, the pore contains a large cationic surface balanced by a large anionic surface (Figure 4D, black arrow pointing to anionic surface). Conversely, PorB also possesses a cationic surface but an anionic surface is reduced in size and polarity (Figure 4E, black arrow pointing to anionic surface). It has been previously proposed that the anionic surface in OmpF drives the cation selectivity through the porin^92^ and this was rationalised as the basis for why primary amines on molecules enhance accumulation in *E. coli*^70^^,^^88^^,^^93^. We observed that the anionic surface patch is reduced in PorB and this is in agreement with previous reports suggesting the charge distribution is less pronounced than that for OmpF^91^. Thus, it stands to reason that the addition of a primary amine, or maintaining a net positive charge, may not be critical to molecule accumulation within *N. gonorrhoeae*. Conversely, the presence of a negative charge may also not be as detrimental to accumulation in *N. gonorrhoeae* as it is for *E. coli*. In fact, it has been reported previously that negatively charged antibiotics, such as penicillin G or cefotaxime, are capable of traversing PorB^94^. Moreover, the current drug of choice for treating *N. gonorrhoeae*, ceftriaxone, is marketed as a di-sodium salt possessing a net charge of −2, yet is still capable of reaching its intracellular target.

**Figure 5.**
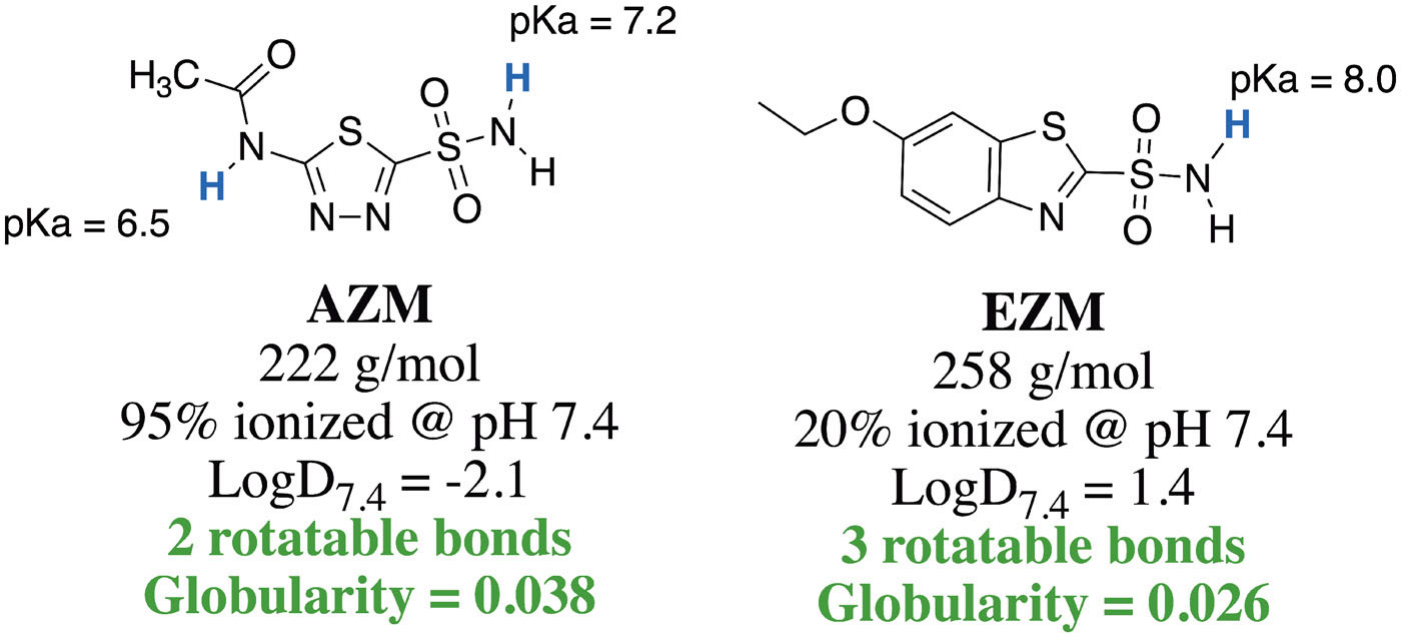
Physicochemical and eNTRy rules properties for AZM and EZM. Green text indicates favourable properties for *E. coli* accumulation defined by Richter et al.^70^ and calculated by entry-way.org.

Both AZM and EZM are negatively ionised at pH 7.4, although to different degrees. The pKa of the sulphonamides vary due to the makeup of the heterocyclic ring. AZM possesses two acidic protons, the amide proton with a predicted pKa of 6.5 and the sulphonamide that has an experimentally determined pKa of 7.2^95^. These combined pKa’s indicate that AZM would be approximately 95% negatively ionised at pH 7.4 (MarvinSketch prediction, Ver 21.8, ChemAxon). Conversely, EZM possesses only one acidic proton on the sulphonamide with a pKa of 8.0^96^ that would yield 20% negatively ionised molecule in solution at pH 7.4. Additionally, EZM is more lipophilic with a predicted LogD_7.4_ of 1.4 compared to a LogD_7.4_ of −2.1 for AZM (MarvinSketch prediction, Ver 21.8, ChemAxon). In terms of molecular weight, globularity, and rigidity, both AZM and EZM are comparable (Figure 5). Thus, given that the interior of PorB is more hydrophobic in nature compared to the OmpF core, we hypothesise that the lower percentage of ionised molecule and increased lipophilicity of EZM may facilitate its improved accumulation within the *N. gonorrhoeae* bacterial cell. This analysis does not account for the effect that these physicochemical properties may have on drug efflux and future studies are planned to investigate the effect of physicochemical properties of molecules on accumulation in *N. gonorrhoeae.*

Extending the analysis, the combination of improved intracellular accumulation and tight binding affinity is likely contributing to the prolonged post-antibiotic effects of EZM. Once EZM is within the cell, the double-digit nanomolar *K*_i_ value for EZM to NgCA would contribute to keeping the molecule inside the bacterial cell as the drug would spend more time in a bound state as opposed to unbound that is subject to diffusion or expulsion out of the cell. Consequently, these properties of EZM could lead to providing the antimicrobial effect for extended periods even after the drug has been washed away, resulting in prolonged PAE. However, the accumulation study was only performed throughout 2 h while the PAE was observed up to 10 h. Future experiments will investigate bacterial accumulation at time points beyond 2 h to determine if and when intracellular EZM levels begin to regress towards baseline as well as evaluate physicochemical properties that may improve accumulation in *N. gonorrhoeae* and how those might differ from *E. coli*.

It is also important to assess the frequency of development of resistance against novel antibacterial agents as an important step in the drug development process. Thus, we attempted to generate *N. gonorrhoeae* mutants that are resistant to either AZM or EZM using a single-step spontaneous mutation assay. At a high inoculum size (∼10^9^ CFU/mL), *N. gonorrhoeae* mutants exhibiting resistance to either AZM or EZM could not be isolated. This indicates a low likelihood of the emergence of rapid resistance to CAIs although further work is being performed on this front. On the other hand, resistance was developed against rifampicin, which is consistent with previous reports^58–60^^,^^77^.

The possibility of targeting the bacterial carbonic anhydrase for anti-gonococcal activity is further bolstered by this new data. Although EZM is FDA-approved, much less is reported in terms of the drugs *in vivo* pharmacokinetics and safety. These attributes would need to be further explored to realise the potential of EZM-based molecules as new antibiotics. Nonetheless, the combined potent antimicrobial activity, extended post-antibiotic effect, and low potential of resistance development towards EZM suggest the scaffold is worthy of additional evaluation as a potential new class of inhibitors to treat drug-resistant *N. gonorrhoeae*.

## 5. Conclusion

In this study, our group has characterised the anti-gonococcal activity for two FDA-approved carbonic anhydrase inhibitors, AZM and EZM. Building from previous reports and work from our team, we have shown that EZM displays a potent antimicrobial activity against *N. gonorrhoeae* clinical isolates with an MIC_50_ value of 0.125 µg/mL and MIC_90_ value of 0.25 µg/mL, which was 16-fold more potent than AZM. EZM also outperformed azithromycin in most strains and was about 4-fold less active than the current drug of choice, ceftriaxone. Consistent with our previous report with AZM-based analogs, we observed that EZM was bacteriostatic against the strains tested and likely inhibits the intracellular *N. gonorrhoeae* carbonic anhydrase as the mode of action. Both AZM and EZM displayed comparable sub-100 nM *K*_i_ values against NgCA. Additionally, it was shown that EZM accumulated within *N. gonorrhoeae* bacteria up to 3.5-fold better than AZM and was hypothesised that reduced ionisation, combined with higher lipophilicity, may enhance accumulation in this particular Gram-negative pathogen. The significant increase in intracellular accumulation likely explains both the superior activity and the extended post-antibiotic effect observed for EZM. Furthermore, AZM and EZM exhibited low potential for the development of bacterial resistance against them. Taken together, the data presented suggests the potential for EZM-based bacterial CAIs that warrant further investigation for translation into effective anti-*N. gonorrhoeae* agents.

## Supplementary Material

Supplemental Material
