# Supplementary material for: Repurposing FDA-approved sulphonamide carbonic anhydrase inhibitors for treatment of Neisseria gonorrhoeae
Source: J Enzyme Inhib Med Chem. 2021 Dec 11;37(1):51–61. doi: 10.1080/14756366.2021.1991336 (PMC8667909; doi:10.1080/14756366.2021.1991336)
Supplement: Supplemental Material [file IENZ_A_1991336_SM8024.pdf]

Supporting Information

Repurposing studies of FDA-approved sulfonamide carbonic anhydrase inhibitors for treatment of *Neisseria gonorrhoeae*

Nader S. Abutaleb[a], Ahmed E. M. Elhassanny[a], Alessio Nocentini[b], Chad S. Hewitt[c], Ahmed Elkashif [d], Bruce R. Cooper[e], Claudiu T. Supuran[b], Mohamed N. Seleem[a,f,], Daniel P. Flaherty[c,g,h]*.*

[a] *Department of Biomedical Sciences and Pathobiology, Virginia-Maryland College of Veterinary Medicine, Virginia Polytechnic Institute and State University, Blacksburg, Virginia, United States.*

[b] *Department of NEUROFARBA, Section of Pharmaceutical and Nutraceutical Sciences, University of Florence, Polo Scientifico, Firenze, Italy.*

[c] *Department of Medicinal Chemistry and Molecular Pharmacology, College of Pharmacy, Purdue University, West Lafayette, Indiana, United States.*

[d] *Department of Comparative Pathobiology, College of Veterinary Medicine, Purdue University, West Lafayette, Indiana, United States.*

[e] *Metabolite Profiling Facility, Bindley Bioscience Center, Purdue University, West Lafayette, Indiana, United States.*

[f] *Center for Emerging, Zoonotic and Arthropod-borne Pathogens, Virginia Polytechnic Institute and State University, Blacksburg, Virginia, United States.*

[g] *Purdue Institute for Drug Discovery, West Lafayette, Indiana, United States.*

[h] *Purdue Institute of Inflammation, Immunology and Infectious Disease, West Lafayette, Indiana, United States.*

Corresponding Authors

Daniel P. Flaherty, PhD
575 Stadium Mall Dr.
West Lafayette, IN, USA, 47907
ORCID: orcid.org/0000-0002-8305-0606
Email: dflaher@purdue.edu

Mohamed N. Seleem, PhD
1410 Prices Fork Rd.
Blacksburg, VA, USA, 24061
ORCID: orcid.org/0000-0003-0939-0458
Email: seleem@vt.edu

Table S1. *N. gonorrhoeae* strains used in the study

***N. gonorrhoeae* strains**	**Description**
CDC 165	Resistant to tetracycline, penicillin, and ciprofloxacin
CDC 166	Resistant to tetracycline, penicillin, and ciprofloxacin
CDC 167	Resistant to azithromycin
CDC 168	Resistant to tetracycline, penicillin, and ciprofloxacin
CDC 169	Resistant to tetracycline, penicillin, and ciprofloxacin
CDC173	Resistant to tetracycline, ciprofloxacin and penicillin
CDC 178	Resistant to tetracycline, penicillin, and ciprofloxacin
CDC 179	Resistant to azithromycin
CDC 181	Resistant to azithromycin and tetracycline
CDC 182	Resistant to tetracycline, ciprofloxacin and penicillin
CDC 183	Resistant to tetracycline, ciprofloxacin and penicillin
CDC 184	Resistant to tetracycline, ciprofloxacin and penicillin
CDC 186	Resistant to tetracycline, ciprofloxacin and penicillin
CDC 187	Resistant to penicillin
CDC 197	Resistant to tetracycline, penicillin, and ciprofloxacin
CDC 202	Resistant to azithromycin
CDC 211	Resistant to tetracycline, penicillin, and ciprofloxacin
ATCC 700825	Isolated from male patient with disseminated gonococcal infection in 1983<br>Resistant to streptomycin
WHO-V	Isolated from a urethritis patient, Sweden, 2012
WHO-W	Isolated in Hong Kong, 2007<br>Resistant to tetracycline, ciprofloxacin and penicillin
WHO-X	Isolated in Japan, 2009<br>Resistant to tetracycline, ciprofloxacin and penicillin<br>Reduced susceptibility to ceftriaxone and cefixime
WHO-Z	Isolated from a female patient with genital infection. Australia, 2013<br><br>Penicillin, tetracycline and ciprofloxacin-resistant<br><br>Reduced susceptibility to ceftriaxone and cefixime

Table S2. MICs (µg/mL) against clinical *Neisseria gonorrhoeae* isolates in CO_2 and ambient Non-CO_2 conditions.

***N. gonorrhoeae* strains**	**Ethoxzolamide**		**Acetazolamide**		**Azithromycin**		**Ceftriaxone**	
	**CO_2**	**Non-CO_2**	**CO_2**	**Non-CO_2**	**CO_2**	**Non-CO_2**	**CO_2**	**Non-CO_2**
CDC 165	>64	0.25	>64	4	2	1	0.06	0.06
CDC 166	>64	0.125	>64	4	1	1	0.125	0.125
CDC 167	64	0.25	>64	2	8	4	0.015	0.015
CDC 168	64	0.125	64	4	1	1	0.125	0.125
CDC 169	>64	0.125	>64	4	1	1	0.125	0.125
CDC 173	64	0.06	>64	1	1	1	0.125	0.125
CDC 178	>64	0.125	>64	2	1	1	0.03	0.03
CDC 179	>64	0.125	>64	4	8	4	0.008	0.008
CDC 181	64	0.25	>64	2	>64	>64	0.015	0.015
CDC 182	64	0.125	32	4	1	1	0.03	0.03
CDC 183	32	0.06	32	2	1	1	0.06	0.03
CDC 184	32	0.06	64	2	1	0.5	0.06	0.06
CDC 186	64	0.125	64	2	0.5	0.5	0.06	0.03
CDC 187	64	0.125	64	2	2	2	0.015	0.015
CDC 197	>64	0.25	64	4	2	2	0.015	0.015
CDC 202	>64	0.25	>64	4	16	16	0.008	0.008
CDC 211	64	0.25	64	1	2	2	0.03	0.03
ATCC 700825	>64	0.125	64	0.5	0.125	0.125	0.008	0.008
WHO-V	32	0.015	>64	0.5	16	16	0.008	0.008
WHO-W	>64	0.25	>64	2	0.25	0.125	0.06	0.06
WHO-X	>64	0.25	>64	1	0.25	0.25	0.5	0.5
WHO-Z	>64	0.5	>64	4	2	2	0.5	0.5
**MIC_{50}**	64	0.125	>64	2	1	1	0.03	0.03
**MIC_{90}**	>64	0.25	>64	4	16	16	0.125	0.125

MIC_{50}: minimum inhibitory concentration at which the compound/drug inhibited 50% of the tested strains. MIC_{90}: minimum inhibitory concentration at which the compound/drug inhibited 90% of the tested strains.

Table S3. Drug accumulation values in *N. gonorrhoeae* ATCC700825

	**Accumulation (nmol/10^9 CFUs)**			
	**10 min**	**30 min**	**60 min**	**120 min**
**Tetracycline**	12.5 ± 3.8	7.9 ± 1.2	5.0 ± 0.8	5.5 ± 0.3
**AZM**	4.3 ± 0.3	5.8 ± 0.1	7.5 ± 0.4	7.7 ± 3.0
**EZM**	17.3 ± 2.9	21.1 ± 2.2	20.3 ± 4.7	19.7 ± 4.0

Values represent average from two biological replicates ± S.D. Each sample contained a dose of 50 nmol/10^9 CFUs for each compound.
